# Planetary Milling and Self-Propagating High-Temperature Synthesis of Al-TiB_2_ Composites

**DOI:** 10.3390/ma13051050

**Published:** 2020-02-26

**Authors:** Alexey Matveev, Ilya Zhukov, Mansur Ziatdinov, Alexander Zhukov

**Affiliations:** National Research Tomsk State University, 36, 634050 Tomsk, Russia; gofra930@gmail.com (I.Z.); ziatdinovm@mail.ru (M.Z.); zhuk_77@mail.ru (A.Z.)

**Keywords:** planetary milling, self-propagating high-temperature synthesis, temperature, combustion, cermet composites, structure, phase composition, titanium diboride, aluminum

## Abstract

The paper explores the influence of planetary milling on the temperature and velocity of Al-Ti-B powder mixture combustion and also on the structure and phase composition of the reaction products. It is found that the time increase of planetary milling modifies the structure of the powder particles, improves the density of compacted specimens, and increases the temperature and velocity of their combustion. These time dependences are extreme, with maximum values during 180 s planetary milling. Experiments show that the reaction products consist of an aluminum matrix with uniformly distributed particles of titanium diboride of not over 1 µm in size. The average particle size changes with the increase in the time of the planetary milling of the initial powder mixture.

## 1. Introduction 

Al-TiB_2_ composites obtained by self-propagating high-temperature synthesis (SHS) or combustion synthesis of Al-Ti-B powder mixture have a high potential to be applied in automobile and aerospace industries [1,2,3]. Moreover, as is known, titanium diboride (TiB_2_) particles are effective nucleators, allowing the fabrication of products with a fine-grain structure [4,5,6,7,8,9,10]. In previous research [9], we show that the addition of 1 wt.% TiB_2_ in the melt of the grade AD35 aluminum alloy reduces the particle size in the fabricated products from 620 to 220 µm and increases their ultimate tensile strength from 100 to 145 MPa.

A control for the structure and phase composition of Al-TiB_2_ composites assists in modifying their physical and mechanical properties and the structure and properties of aluminum (Al) alloys when using them as master alloys.

Self-propagating high temperature synthesis (SHS) is synthesis which occurs through the exothermic reaction of two or more components with the release of more heat, which allows the synthesis reaction in autowave mode to be maintained. In the process of this reaction, new materials are formed with fundamentally new properties. It is well known that a change in the SHS parameters, viz. the combustion temperature and velocity, modifies the structure of obtained materials [8]. It is shown [9,10] that, with an increasing content of Al powder in the initial Al-Ti-B powder mixture, the temperature and the velocity of combustion are reduced, resulting in TiB_2_ particle size reduction in the obtained materials. 

In [11,12,13,14], it was reported that planetary milling significantly improved the exothermic nature of the initial powder mixture. According to that research, the planetary milling of Ti + 2B, Ni + Al, Ti + C and Ti-Al powder mixtures changed the particle structure of compositions and caused the formation of composite particles comprising layers of the initial components. It was also shown that planetary milling increased the combustion temperature and velocity and lowered the initiation temperature of the reaction [12].

The planetary milling of Al-Ti-B powder mixture can therefore be expected to change the combustion temperature and velocity as well as the structure and phase composition of the reaction products.

The aim of this work is to study how the time of the planetary milling of Al-Ti-B powder mixture (60 wt.% Al + 40 wt.% (Ti + 2B)) changes the combustion temperature and velocity and the structure and phase composition of the reaction products. 

## 2. Materials and Methods

The composite specimens were produced from a mixture of aluminum, titanium, and boron powders, the average particle sizes of which are given in Table 1.

These components were mixed in ethanol in the following proportion: 60 wt.% Al; 27.6 wt.% Ti; 12.4 wt.% B (65 vol.% Al; 18.18 vol.% Ti; 16.00 vol.% B). The mixture was dried at 80 °C in a vacuum chamber and then placed in a drum with steel balls for planetary milling in a planetary mill. The mass ratio of the steel balls and the mixture was 20:1. The diameter of steel balls was 0.5 сm. Planetary milling was performed with a 50 g (840 rpm) load; its duration was varied from 60 to 2400 s. The obtained powder mixture was compressed to produce specimens with a diameter of 2.3 cm and 25 g weight. Compression was conducted under a pressure of 215 MPa. Further density was measured on these compressed pellets. The SHS procedure was described in our previous research [9,15]. Tungsten-rhenium thermocouples were used for the measurements of combustion temperature [16]. The burning rate was determined as the length of the sample divided by the burning time. The process of combustion was recorded on a video camera.

The phase composition of specimens was obtained using a Shimadzu XRD-6000 diffractometer (Kyoto, Japan), equipped with Ni filtered Cu Kα as the X-ray source. The Powder Diffraction File (PDF-4) database of the International Centre for Diffraction Data, Denver, USA was used to compare the diffraction peaks of the obtained X-ray diffraction (XRD) patterns.

A Philips SEM 515 scanning electron microscope (SEM, Amsterdam, Netherlands) and an EDAX Genesis microanalyzer, Netherlands was used to investigate the structure of the SHS products, which were processed on a polishing machine. The polished specimen surface was then etched in a 10% HCl solution. 

Elemental analysis was performed by energy dispersive X-ray spectroscopy.

The medium sizes of the ceramic particles in the SHS products were determined from SEM images in a 300 Image Tool program using the secant method [17,18].

## 3. Results

### 3.1. Planetary Milling Time vs. Structure and Density of Al-Ti-B Powder Mixture 

**Figure 1:**
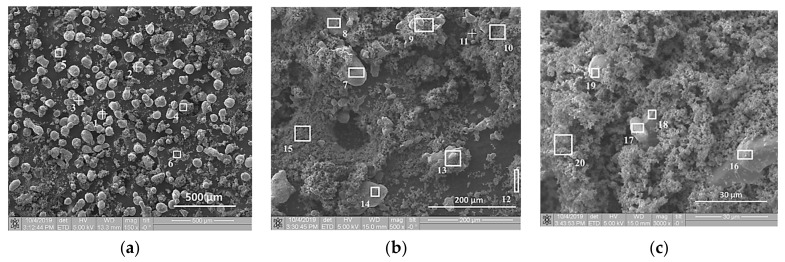
Scanning electron microscope (SEM) images of Al-Ti-B powder particles before planetary milling at different magnifications: (**a**) 150×; (**b**) 500×; (**c**) 3000×.

**Figure 2:**
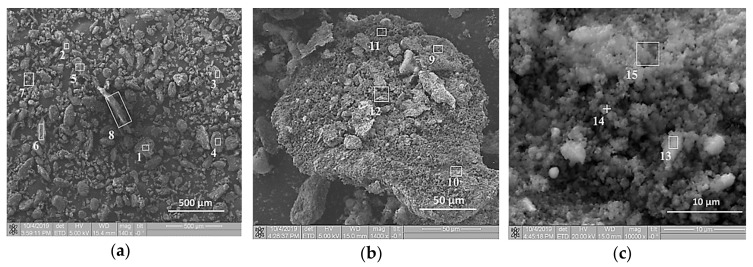
SEM images of Al-Ti-B powder particles after 900 s planetary milling at different magnifications: (**a**) 140×; (**b**) 1400×; (**c**) 10000×.

**Figure 3:**
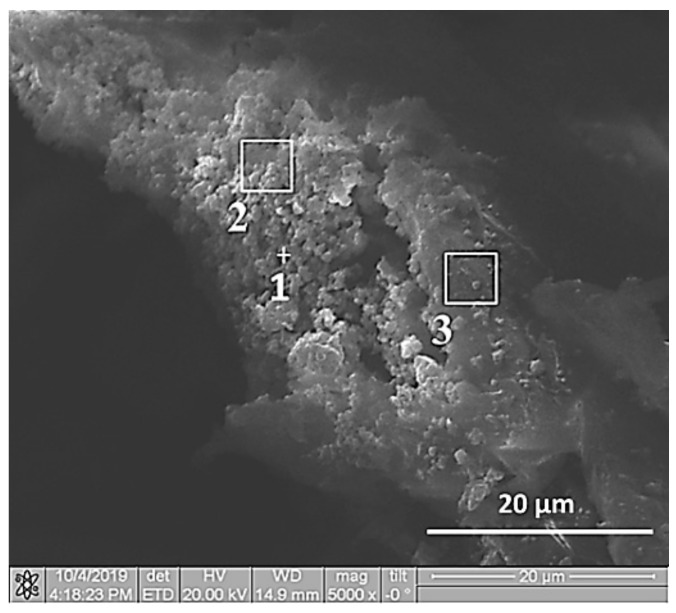
SEM image of Al-Ti-B composite particle.

SEM images of the initial structure of the Al-Ti-B mixture are presented in Figure 1 and Figure 2 before and after planetary milling for 900 s. In Figure 3, the SEM image indicates the region 8 in Figure 2а, which shows the particle cross-section. Table 2, Table 3 and Table 4 summarize the results of the elemental analysis of the mixture and the particle cross-section before and after planetary milling. Before planetary milling, the powder mixture comprises individual Al and Ti particles with B particles on their surface. As Figure 1b,c shows, the main boron content is concentrated on the surface of Ti particles, and only isolated B particles are observed on the surface of Al particles. After planetary milling, the particle structure changes; i.e., the deformation and fragmentation of Al and Ti particles occur. The resulting plate-like composite particles presented in Figure 2b,c and Figure 3 consist of Al, Ti, and B particles with sizes less than 1 μm. 

**Figure 4:**
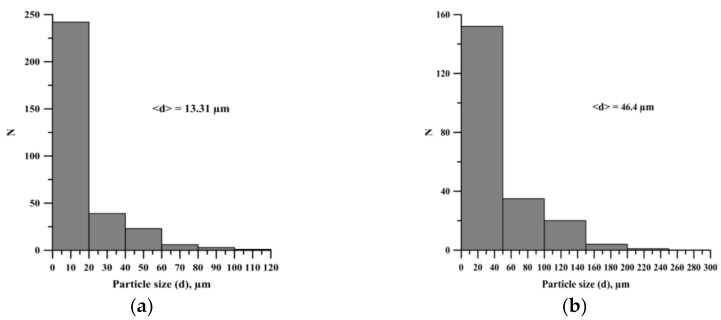
Histogram of particle size distribution: (**а**) before planetary milling, (**b**) after 900 s planetary milling.

**Figure 5:**
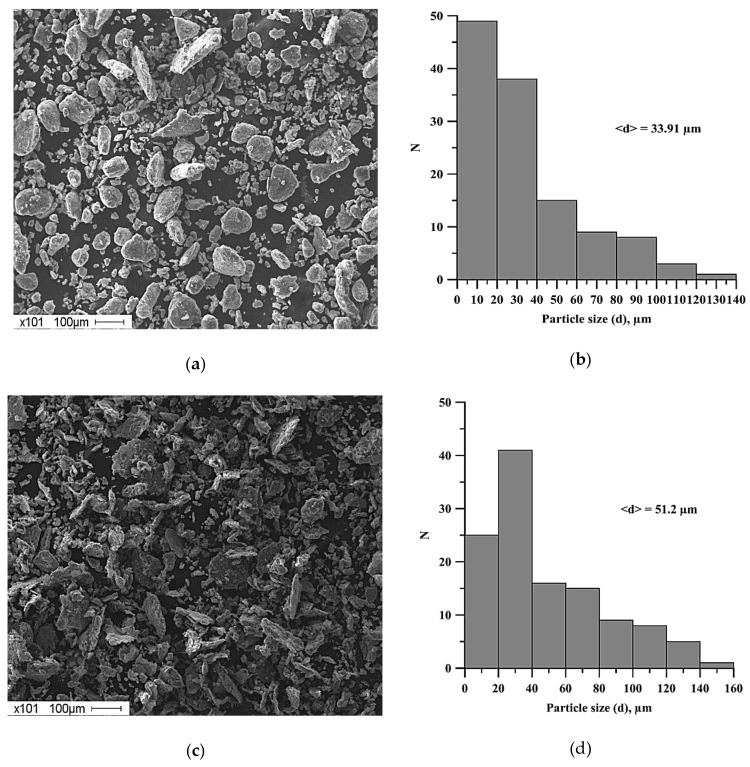
SEM images of the structures of powder mixtures of Al-Ti-B and histograms of the distribution of particles of the mixtures: (**a**)-(**b**) after 180 s planetary milling, (**c**)-(**d**) after 2400 s planetary milling.

The average particle size, obtained after the planetary milling of the mixture, is larger compared to the average particle size of the original mixture (Figure 4). An increase in the time of planetary milling leads to the formation of large agglomerates and an increase in the average particle size of the powder mixture (Figure 5).

**Figure 6:**
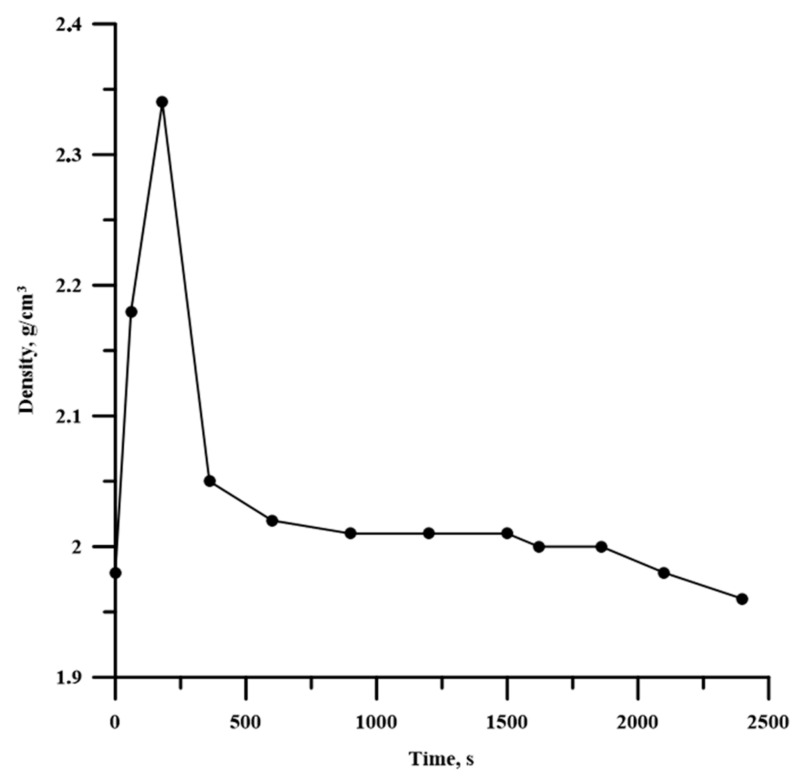
Dependence between the time of planetary milling and the density of the Al-Ti-B powder mixture.

We assumed that the particle deformation affected the compaction process of the powder mixture. Actually, when changing the time of planetary milling, the specimen density also changed, and this dependence is extreme; it is given in Figure 6. 

### 3.2. Time Dependence of Planetary Milling of the Initial Al-Ti-B Powder Misxture on Its Combustion Temparature and Velocity 

The combustion of the initial (not planetary milled) Al-Ti-B powder mixture failed. After planetary milling for a period from 60 to 900 s, the combustion process was initiated. A spin combustion wave propagated over the whole specimen [19,20], without attenuation. 

**Figure 7:**
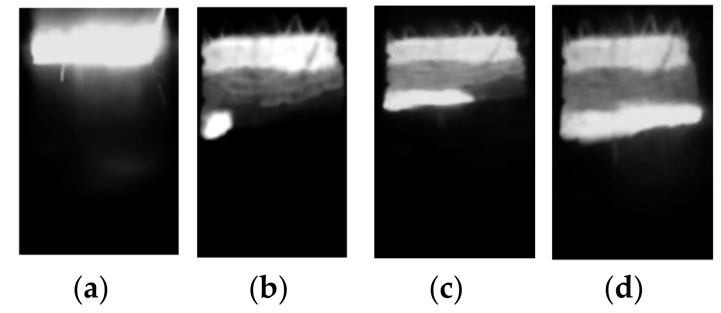
Images of typical spin combustion synthesis: (**а**) ignition; (**b**) spin wave nucleation; (**c**) spin wave motion; (**d**) spin wave completion.

A typical SHS process is illustrated in Figure 7. A further increase in planetary milling up to 2400 s resulted in combustion attenuation.

**Figure 8:**
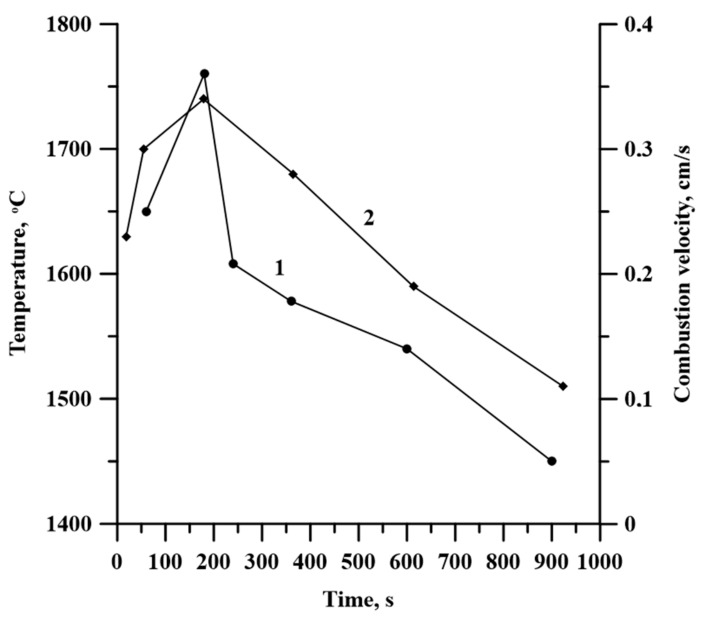
Time curves of combustion temperature (1) and velocity (2).

Figure 8 contains the plot of the combustion temperature and velocity depending on the time of mechancal activation. These are extreme dependencies, which correlate with each other and with the specimen density.

### 3.3. Time Dependence of Planetary Milling of the Al-Ti-B Powder Misxture on the Phase Composition and Structure of Combustion Products

**Figure 9:**
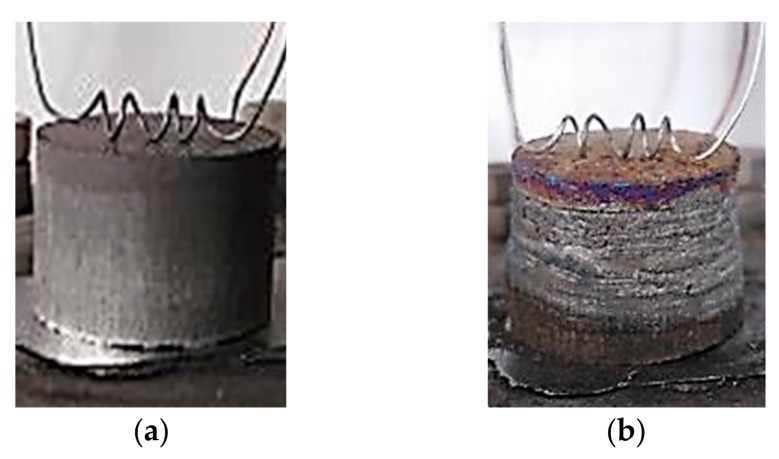
Photographs of Al-Ti-B specimen: (**a**) before combustion; (**b**) after combustion synthesis.

The initial specimen, a photograph of which is presented in Figure 9а, is a cylindrical pellet of a gray metallic color. The combustion product in Figure 9b represents a fused specimen of a metallic color. Its surface is cracked, with visible metal layers. The length and the diameter of the obtained product increase by 15.5% and 7.4%, respectively.

**Figure 10:**
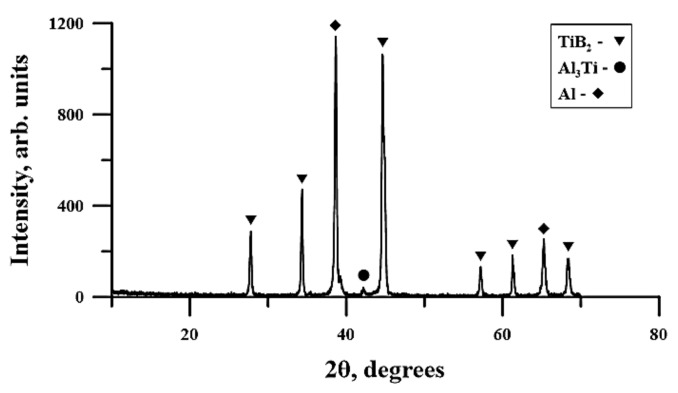
Typical X-ray diffraction (XRD) pattern of the planetary milled Al–Ti–B-based combustion product.

A typical XRD pattern of the planetary milling combustion product obtained from the powder mixture is presented in Figure 10. The results of the XRD analysis are gathered in Table 5.

According to Table 5, the SHS products do not qualitatively differ from each other and contain TiB_2_, Al, and traces of the Al_3_Ti phase. The lattice parameters in all the specimens differ insignificantly. One can see a change in the phase composition and the size of the coherent scattering region with an increasing time of planetary milling. The coherent scattering region in titanium diboride and aluminum is extreme; viz. 97 and 100 nm, respectively. The coherent scattering region is a characterized region of crystallite that scatters X-rays coherently and independently of other similar regions. The CSR size is used to analyze the size of crystallites in polycrystals and is measured experimentally with the help of diffraction reflection attenuation.

**Figure 11:**
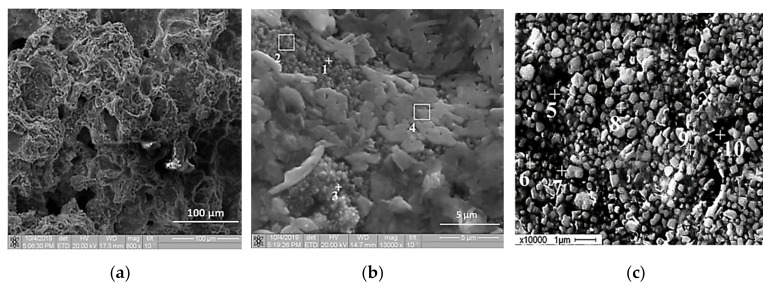
SEM images of self-propagating high-temperature synthesis (SHS) product: (**a**), (**b**) cross-section; (**c**) polished and etched surface.

Figure 11 presents SEM images of the specimen structure obtained after the Al-Ti-B mixture combustion synthesis. The results of the elemental analysis of the combustion products are summarized in Table 6. According to Figure 11а, the specimen structure is porous, and in Figure 11b, one can see particles which are not larger than 1 µm and plate-like particles in the shape of a flower. Unlike other particles which consist of Al, Ti, and B elements, plate-like particles in the shape of a flower consist of Ti and Al. Figure 11c shows the surface of the obtained product after polishing and etching. There are well-defined particles which are uniformly distributed in the matrix.

**Figure 12:**
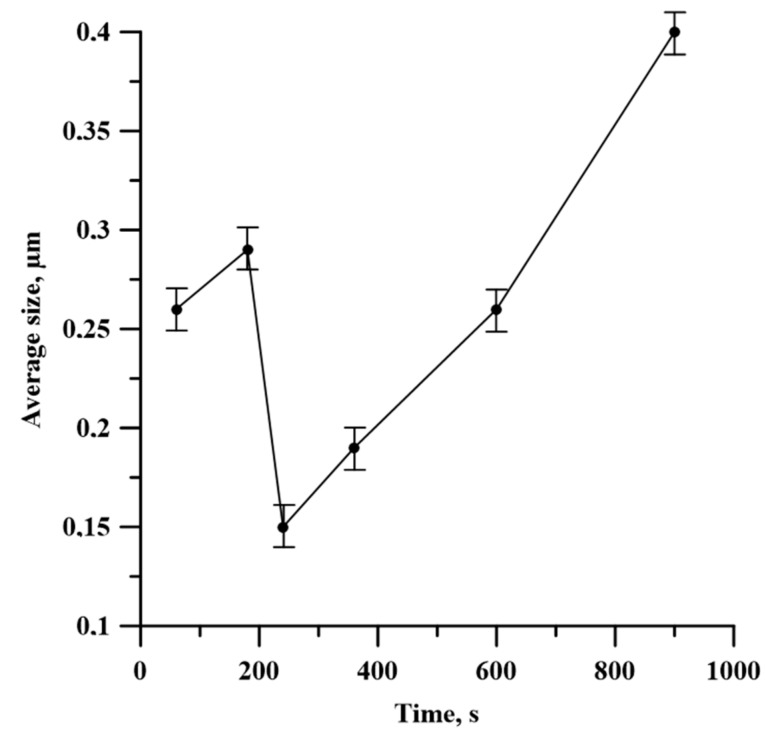
Time dependence of Al_3_Ti–TiB_2_ particle size on planetary milling.

Figure 12 describes the average size of TiB_2_ particles in the SHS product depending on the time of the planetary milling of the Al-Ti-B powder mixture. It is found that when the time of planetary milling is increased from 60 to 180 s, the average particle size grows from 0.26 to 0.29 µm in the obtained SHS products. After the further planetary milling of the powder mixture within the range from 180 to 240 s, the average particle size decreases from 0.29 to 0.15 µm. Finally, the increase in planetary milling from 240 to 900 s provides a growth in the average particle size up to 0.4 µm.

## 4. Discussion

After planetary milling of the Al-Ti-B powder mixture, Al and Ti particles become smaller, flatten, and, combining with B particles, form composites with Al, Ti, and B inclusions [13,14]. As the mixture homogenization occurs, the reacting area and the specimen density increase. These processes lead to the temperature elevation and the growth in the combusion velocity with an increasing time of planetary milling [11,12]. Moreover, during planetary milling, the energy accumulates inside composite particles due to their stress–strain state caused by the rotation of steel balls. The accumulated energy also promotes the increase in the combustion temperature and velocity [21,22,23,24]. Planetary milling longer than 180 s leads to the formation of large agglomerates and an increase in the average particle size of the mixture. This leads to a reduction in the reacting surface and the density of the specimens, thereby decreasing the combustion temperature and velocity.

The formation of pores, defects, and cracks on the specimen surface as well as the change in the specimen size result from the release of various impurities (B_2_O_3_, O_2_, and others) from the specimen bulk during the combusion process.

In Table 7, we gather the standardized lattice parameters of Al, Al_3_Ti, and TiB_2_ phases [25,26,27,28].

A comparison of the values presented in Table 7 and Table 5 shows that the lattice parameters of phases found in the combusion product of the Al-Ti-B mixture do not significantly differ from the standardized data.

The extreme dependence of grains inside Al and TiB_2_ particles is conditioned by the change in the combusion temperature which affects the growth of these grains [29]. Thus, 180 s planetary milling provides the highest combusion temperature of the powder mixture and the highest grain size in the particles.

**Figure 13:**
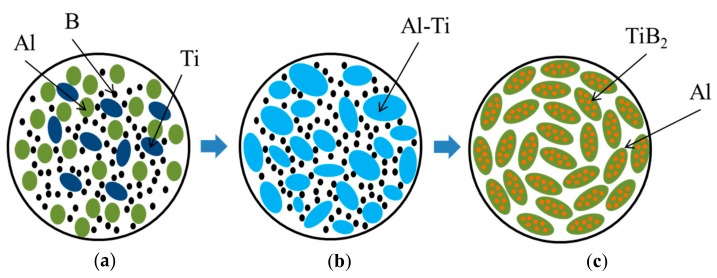
Schematic of structure formation in Al-TiB_2_-Al_3_Ti composite: (**a**) initial powder mixture; (**b**) melting of Al particles and melt distribution between Ti and B particles; (**c**) combustion product.

According to the XRD and elemental analyses of the local areas of the specimen structure, we assume that the combustion products consist of the metallic Al matrix and TiB_2_ particles uniformly distributed in it. The XRD pattern also shows trases of the Al_3_Ti phase. Similar results were obtained by Fan, et al. [5], who synthesized Al–TiB_2_ composites through the introduction of Al particles in the Ti–B powder mixture. They detected an intermetallic layer of Al_3_Ti between the TiB_2_ particles and the Al matrix. The authors in [5] explained the formation of the Al_3_Ti phase on the surface of TiB2 particles: the aluminum melt adsorbs part of the titanium particles from the surface of titanium diboride. Based on the experimental findings, we propose a mechanism of the structure formation in the combusion products obtained; considering this process, the schematic is shown in Figure 13.

During the spin wave propagation, Al particles begin to melt (see Figure 13а) and distribute between Ti and B particles. In the Al melt, these particles dissolve. As presented on the state-transition diagrams for Ti-Al and Al-B mixtures, given in the works [30,31,32], the dissolution of titanium in aluminum is much higher, than that of boron. This is probably because titanium dissolves in liquid aluminum during the combustion process and forms a melt comprising ~68.5 wt.% Al and 31.5 wt.% Ti (Figure 13b). This melt composition matches the composition of 84.7 wt.% Al_3_Ti and 15.3 wt.% Al(liq.) [31]. Boron dissolves in the generated Al-Ti melt. Upon reaching the ultimate boron dissolution, the TiB_2_ phase starts to form with the release of particles ranging between 0.01 and 1 µm in size. The obtained composites uniformly distribute in the Al matrix (Figure 13c). The proposed mechanism of the structure formation in the synthesis products is in agreement with the experimental data [33] on the mixture of Al-8Ti and Al-4B melts.

The dependence between the average size of TiB_2_ particles and the time of planetary milling is in agreement with the dependence between the duration of planetary milling and the size of the coherent scattering region in the TiB_2_ phase, and this is an extreme dependence. From the work of Merzhanov and Mukas’yan [20], it is known that the change in the combustion temperature and velocity affects the structure formation in composite materials, including the size of ceramic inclusions. Therefore, a change in the average particle size of titanium diboride can be explained by the change in the temperature and velocity of Al-Ti-B mixture combustion after its planetary milling.

## 5. Conclusions

The influence of planetary milling on the temperature and combustion velocity of the Al-Ti-B powder mixture, as well as on the structure and phase composition of the reaction products was investigated.

1. Lamellar composites were formed after planetary milling of the Al-Ti-B mixture. These composites were composed of Al, Ti, and B particles less than 1 µm. An increase in the planetary milling time from 180 to 2400 s led to an increase in the average size of the lamellar composite from 33.9 to 51.2 μm.
2. An increase in the time of planetary milling led to a change in the density of the compacted samples, as well as the temperature and their combustion velocity. These time dependences were extreme, with maximum values during 180 s of planetary milling. An increase in the planetary milling time of more than 180 s led to a decrease in the density of compacted samples, as well as the temperature and their combustion velocity. A change in the combustion rate and temperature was associated with a change in the particles structure of the mixture after its planetary milling. The combustion of samples obtained after planetary milling of the mixture was carried out in the spin combustion mode.
3. The structure of SHS-materials obtained from Al-Ti-B powder systems after planetary milling was a metal matrix Al with inclusions of TiB_2_ particles. The Al_3_Ti layer was discovered between the TiB_2_ and the aluminum matrix. The TiB_2_ particle size was less than 1 μm in all samples. The dependence of the average size of TiB_2_ particles in SHS products on the duration of planetary milling of the initial mixture was established. The increasing planetary milling time from 60 to 180 s, the average particle size in the obtained SHS products increased from 0.26 to 0.29 μm. After further planetary milling of the powder mixture in the range from 180 to 240 s, the average particle size decreased from 0.29 to 0.15 µm. Finally, an increasing in planetary milling from 240 to 900 s ensured an increase in the average particle size to 0.4 μm.
4. The formation mechanism of Al-Al_3_Ti-TiB_2_ composites during combustion of the Al-Ti-B powder mixture in the mode of self-propagating high-temperature synthesis was established.

## Figures and Tables

**Table 1:** Initial powders.

Powder	Average Particle Size, µm	Purity, wt. %
Titanium (Ti) (OJSC «Polema»)	140	⩾99
Boron (B) (OJSC «Aviabor»)	0.6	⩾99.2
Aluminum (Al) (Ltd «Sual-PM»)	100	⩾98.4

**Table 2:** Quantitative elemental analysis of Al-Ti-B powder particles before planetary milling («+»discovered, «-» not discovered).

**Element**	**1**	**2**	**3**	**4**	**5**	**6**	**7**	**8**	**9**	**10**
Al	+	+	+	+	+	+	+	+	+	+
Ti	-	-	+	+	+	+	+	-	-	+
B	-	-	-	+	+	-	+	+	-	+
**Element**	**11**	**12**	**13**	**14**	**15**	**16**	**17**	**18**	**19**	**20 ^1^**
Al	+	-	-	+	+	+	-	+	-	+
Ti	+	+	+	-	+	+	+	-	+	+
B	+	+	+	-	+	+	-	-	-	+

^1 ^Observation areas (Figure 1).

**Table 3:** Quantitative elemental analysis elemental analysis of Al-Ti-B powder particles after 900 s planetary milling.

Element	1–2	3	4–15 ^1^
Al	+	+	+
Ti	+	-	+
B	+	+	+

^1^ Observation areas (Figure 2).

**Table 4:** Quantitative elemental analysis elemental analysis of Al-Ti-B powder particle cross section after 900 s planetary milling.

Element	1	2	3 ^1^
Al	+	+	+
Ti	+	+	+
B	+	+	+

^1^ Observation areas (Figure 3).

**Table 5:** XRD-analysis results of synthesized products based on the planetary milled Al-Ti-B mixture.

Specimens	Phases	Composition, wt.%	Lattice Parameters, Ǻ	CSR ^2^, nm
60 s PM^1^ (Z1_306_19)	TiB_2_	44	*a* = 3.0274, *c* = 3.2261	71
	Al	55	*a* = 4.0455	68
	Al_3_Ti	Traces	*a* = 3.8462, *c* = 8.5904	-
180 s PM (Z1_307_19)	TiB_2_	40	*a* = 3.0290, *c* = 3.2285	97
	Al	58	*a* = 4.0487	100
	Al_3_Ti	Traces	*a* = 3.8504, *c* = 8.5932	-
360 s PM(Z1_308_19)	TiB_2_	42	*a* = 3.0296, *c* = 3.2293	74
	Al	55	*a* = 4.0501	48
	Al_3_Ti	Traces	*a* = 3.8528, *c* = 8.5979	47
900 s PM (Z1_309_19)	TiB_2_	39	*a* = 3.0256, *c* = 3.2264	43
	Al	54	*a* = 4,0447	38
	Al_3_Ti	Traces	*a* = 3.8430, *c* = 8.5854	35

^1^ Planetary milling; ^2^ Coherent scattering region.

**Table 6:** Quantitative elemental analysis elemental analysis of SHS product.

Element	1, 2, 3, 6, 8, 9	4, 5, 6, 7, 10 ^1^
Al	+	+
Ti	+	+
B	+	-

^1^ Observation areas (Figure 9).

**Table 7:** Lattice parameters of Al, Al_3_Ti, amd TiB_2_ phases.

Compound	Lattice Parameters, Ǻ
Al	а = 4.040
Al_3_Ti	а = 3.8467, c = 8.608
TiB_2_	а = 3.026, с = 3.212
